# Changes in Physiopathological Markers in Myotonic Dystrophy Type 1 Skeletal Muscle: A 3-Year Follow-up Study

**DOI:** 10.3233/JND-230139

**Published:** 2024-09-03

**Authors:** Marie-Pier Roussel, Aymeric Ravel-Chapuis, Jonathan Gobin, Bernard J. Jasmin, Jean-Philippe Leduc-Gaudet, Cynthia Gagnon, Elise Duchesne

**Affiliations:** a Département des Sciences Fondamentales, Université du Québec à Chicoutimi, Saguenay, QC, Canada; b Groupe de Recherche Interdisciplinaire sur les Maladies Neuromusculaires (GRIMN), Centre Intégré Universitaire de Santé et de Services Sociaux du Saguenay–Lac-Saint-Jean, Saguenay, QC, Canada; c École des Sciences Pharmaceutiques, Faculté de Médecine, Université d’Ottawa, Ottawa, Canada; d Département de Médecine Cellulaire et Moléculaire, Faculté de Médecine, Université d’Ottawa, Ottawa, Canada; e Centre de Recherche Éric-Poulin sur les Maladies Neuromusculaires, Faculté de Médecine, Université d’Ottawa, Ottawa, Canada; f Groupe de Recherche en Signalisation Cellulaire, Département de Biologie Médicale, Université du Québec à Trois-Rivières, Trois-Rivières, QC, Canada; g Faculté de Médecine et des Sciences de la Santé, Université de Sherbrooke, Sherbrooke, QC, Canada; h École des Sciences de la Réadaptation, Faculté de Médecine, Université Laval, Québec, QC, Canada; i Centre Interdisciplinaire de Recherche en Réadaptation et Intégration Sociale (Cirris), Centre Intégré Universitaire de Santé et de Services Sociaux Capitale-Nationale, Québec, QC, Canada; j CHU de Québec - Centre de Recherche de l’Université Laval, Québec, QC, Canada

**Keywords:** Myotonic dystrophy type 1, natural history study, maximal muscle strength, histomorphology, nuclear foci and MBNL1 colocalization, protein expression

## Abstract

**Background:**

Myotonic dystrophy type 1 (DM1) is a slowly progressive disease caused by abnormal CTG repetitions on the dystrophia myotonica protein kinase (*DMPK*) gene. Long mRNA from CTG repetitions stabilizes in nuclear foci and sequester muscleblind-like splicing regulator 1 (MBNL1). Cardinal signs of DM1 include muscle wasting and weakness. The impacts of DM1 progression on skeletal muscle are under-researched.

**Objective:**

Identifying physiopathological markers related to maximal strength loss over time in DM1.

**Methods:**

Twenty-two individuals with DM1 participated in two maximal isometric muscle strength (MIMS) evaluations of their knee extensors and two *vastus lateralis* muscle biopsies, 3 years apart. Muscle fiber typing, size (including minimal Feret’s diameter [MFD] and atrophy/hypertrophy factors [AF/HF]), and nuclear foci and MBNL1 colocalization (foci/MBNL1+) were evaluated. Immunoblotting was used to measure glycogen synthase kinase-3 beta (GSK3*β*), p62, LC3BI, LC3BII, and oxidative phosphorylation proteins.

**Results:**

There are significant correlations between the fold changes of MIMS with type 1 fiber MFD (*ρ*= 0.483) and AF (*ρ*= –0.514). Regression analysis shows that baseline percentage of foci/MBNL1+ nuclei and strength training explain 44.1% of foci/MBNL1+ nuclei percentage variation over time. There are fair to excellent correlations between the fold changes of MIMS and GSK3*β* (*ρ*= 0.327), p62 (*ρ*= 0.473), LC3BI (*ρ*= 0.518), LC3BII (*ρ*= –0.391) and LC3BII/LC3BI (*ρ*= –0.773).

**Conclusion:**

Type 1 MFD decrease and AF increase are correlated with MIMS loss. There seems to be a plateau effect in foci/MBNL1+ nuclei accumulation and strength training helps decrease this accumulation. Autophagy marker LC3BII/LC3BI ratio has a good biomarker potential of MIMS loss, but more investigations are needed.

## INTRODUCTION

Myotonic dystrophy type 1 (DM1) is a slowly progressive autosomal dominant disease. It is the most prevalent muscular dystrophy in adults [1]. Its worldwide prevalence is 1/20 000 and it reaches its peak in the Saguenay–Lac-St-Jean region of Québec, Canada, where 1/475 individuals are affected [2, 3]. DM1 is a very heterogeneous disease and affects multiple systems, notably the musculoskeletal system, causing muscle atrophy and weakness [1]. It is caused by abnormal cytosine thymine guanine (CTG) repeats in the 3’-untranslated region of the dystrophia myotonica protein kinase (*DMPK*) gene [4]. It is classically divided into five clinical phenotypes according to the severity and the age of onset of the symptoms: congenital, infantile, juvenile, adult (classic) and late-onset [5]. The phenotypes are based partly on the number of CTG repetitions [5]. Another factor that contributes to DM1’s heterogeneous clinical presentation is sex [6]. It has also been shown that men and women present different disease progression [7, 8].

The physiopathology of the disease stems from the CTG repeat expansion that is transcribed in a long cytosine, uracil and guanine (CUG) RNA strand and stabilizes itself in a hairpin formation [9]. These RNA formations accumulate into nuclear foci that become toxic to cells [10, 11]. The gain of function of these nuclear foci is considered the main physiopathological mechanism in DM1 and is the most documented [10–13]. However, this mechanism does not explain the whole clinical presentation of the disease. Efforts are thus made by research groups to better understand the disease by exploring alternative mechanisms [11, 14, 15]. The foci sequester muscleblind-like splicing regulator 1 (MBNL1), an RNA splicing protein, which in turn leads to misplicing events that induce, among other abnormalities, myotonia, insulin resistance and elevated cytoplasmic calcium levels [10, 13]. Another heavily influenced splicing protein is CUGPB elav-like family member 1 (CELF1), which becomes abnormally stabilized and thus experiences a gain of function [10, 11, 13]. The accumulation of foci also leads to an upregulation of staufen-1 (Stau1), protein kinase C (PKC), heterogeneous nuclear ribonucleoprotein A1 (HNRNPA1) and homeobox protein Nkx-2.5 (NKX2-5) [13]. These deregulations promote muscle atrophy (particularly of type 1 fibers) by negatively affecting many signaling pathways and cellular functions, notably the AKT and AMPK pathways, as well as autophagy and apoptosis processes [13]. AKT is a known inhibitor of glycogen synthase kinase-3 beta (GSK3*β*), which has been shown to be overactivated in DM1 and linked to muscle wasting [13]. Furthermore, recent studies have also shown that DM1 muscle has reduced mitochondrial content [16, 17]. Our group also showed that upregulated genes in DM1 myoblasts were enriched for diverse biological functions including autophagy [15]. We also demonstrated that LC3II and p62 protein levels expression were decreased in DM1 suggesting that dysregulated autophagy signaling might contribute to the progression of this disease [18].

The resulting muscle weakness can lead to important physical limitations in individuals with DM1 [19]. Notably, the reduction in maximal strength of the knee extensors is known to be one of the main explanatory factors in the reduced performance of the Timed-up and go test [19]. A longitudinal study has found that over a 9-year period, individuals with DM1 can lose between 24.5% and 52.8% of their maximal strength depending on the evaluated muscle group [7]. In our previous study, we have shown that over a 3-year period, DM1 participants can lose between 30.6% or gain up to 6.0% of their maximal strength depending on the tested muscle group [8]. We have also found that the evaluation of maximal muscle strength with quantified muscle testing is sensitive enough to assess the evolution of the disease [8], while functional tests can miss changes due to the slow evolution of the disease and possible compensations [8]. Therefore, in the context of this study, quantified muscle testing is a relevant proxy to correlate with physiopathological markers. In order to counter muscle weakness, exercise has been shown to be safe [20], and can even result in maximal strength gains [21–24]. Other studies, including our previous paper, also showed that exercise, notably strength training, can have a protective effect on the evolution of muscle weakness in DM1 [8, 25]. Furthermore, a mouse model study has shown that exercise can reduce nuclear foci accumulation and MBNL1 sequestration [26]. Such effect has not yet been demonstrated in humans with DM1 but we hypothesize that strength training is of sufficient intensity to induce this reduction. Most longitudinal studies conducted in DM1 assess clinical outcome measures, such as maximal muscle strength or walking speed [7, 8, 27]. To our knowledge, there are no other studies aimed to evaluate the physiopathological changes in skeletal muscles over time. Such markers are important to identify underlying cellular mechanisms and to evaluate how they evolve with the progression of the disease, and thus inform future clinical trials. These trials could then use this knowledge to assess the effectiveness of an intervention. This study therefore aimed to begin closing this gap in knowledge by identifying physiopathological markers related to the maximal strength loss over time in individuals with DM1.

## METHODS

### Study setting and participants

This present project is a sequel to our previous study showing the clinical progression of DM1 over 3 years [8], and is part of a larger ongoing longitudinal study where the data from phases 3 and 4 (P3 and P4 respectively) were used [7, 8, 28]. Details of the context of this larger longitudinal study, especially for P3 and P4 can be found in the prequel paper Roussel et al. 2021 [8]. A total of 23 participants completed clinical evaluations and have undergone muscle biopsies at P3 and P4. All participants were recruited from the neuromuscular clinic of the *Centre int*é*gr*é *universitaire de sant*é *et de services sociaux (CIUSSS) du Saguenay–Lac-St-Jean (SLSJ)*. The inclusion criteria were 1) to have a genetically confirmed diagnosis of DM1, 2) to be between 18 and 70 years old and 3) to be able to give informed consent. Exclusion criteria were 1) to have any other neuromuscular disease or diseases that have an impact on function (e.g., stroke) and 2) to have any contraindication to a physical evaluation or a muscle biopsy. The project was approved by the committee of ethics of research of the CIUSSS of Saguenay−Lac-St-Jean and a written informed consent was obtained from all participants.

### Procedures

The participants’ characteristics were obtained from research data collected in the larger longitudinal study or from their medical files. All clinical phenotypes were carefully revised by the neuromuscular clinic’s neurologist. Physical evaluations and muscle biopsies were done on separate visits at both time points. All physical evaluations were done before the muscle biopsy to avoid any reduced physical capacities resulting from the post-biopsy healing process. During the physical evaluation visit, participants were measured for height and weighed for body mass. Quantified muscle testing (QMT) of the knee extensors was evaluated as described in Roussel et al. 2019 [29]. The average of the two closest measurements on the side of the biopsied leg was used. Participants were asked what type of physical activity they practiced in the 3 years between the two evaluation time points. Their physical activity type was classified as sedentary, physical activity for any general physical activity and strength training for participants who participated in a training program specifically designed to induce maximal strength gains [8].

#### Muscle biopsies

Muscle biopsies were performed by either of our team’s two general practitioners and they were assisted by trained members of the research team. The skin was disinfected and anesthetized, then a 1 cm incision was made at 15 cm above the patella to access the *vastus lateralis* muscle. Suction-modified Bergström muscle biopsy technique was performed to obtain the muscle sample [30]. The incision was closed by stitches and the wound was covered with a transparent waterproof film dressing. Participants were instructed about proper post-biopsy care and had a follow-up call 48 h post-biopsy. Muscle samples were immediately rinsed in a cold phosphate-buffered saline (PBS) solution and properly processed according to intended lab use. For histology, muscle pieces were frozen in tissue freezing medium put in isopentane that was cooled in liquid nitrogen. For immunoblotting, the muscle pieces were put in a cryovial and flash-frozen in liquid nitrogen. The samples were then stored at –80°C until further use.

#### Immunofluorescence for muscle fiber typing and size

Ten-micrometer thick cross sections of muscle samples were cut at –20°C in a cryostat and placed on positively charged microscope glass slides. Slides were submerged in ice-cold acetone/methanol (60/40) for 10 min for sample fixation. Sections were blocked in PBS with 10% horse serum for 30 min at room temperature and washed in PBS. Primary antibodies (rabbit anti-laminin [Abcam, Cambridge, United Kingdom, 11575, 1:50] and mouse IgM anti-human myosin heavy chain 1 [Developmental Studies Hybridoma Bank, Iowa City, United States, A4.840, 1:250]) were diluted in PBS with 1.5% horse serum and incubated 1 h at room temperature. Sections were rinsed three times in PBS and incubated in secondary antibodies (anti-rabbit 350 [Pierce, Appleton, United States, 62272, 1:50] and anti-mouse IgM 550 [Pierce, Appleton, United States, SA5-1151, 1:1000]) for 1 h at room temperature in the dark. Sections were rinsed three times in PBS and mounted using PermaFluor mounting medium (Thermofisher, Waltham, United States, TA030FM). Immunofluorescence pictures were taken with an Olympus BX61 microscope within 36 hours post-labeling to ensure the best image quality. Pictures were analyzed using ImageJ software (version 5.2). Laminin markings were used to determine muscle fiber size. Myosin heavy chain 1 staining was used to identify type 1 myofibers and unstained myofibers were considered as type 2.

To analyze muscle fiber size, the minimal Feret’s diameter (MFD) was used since it is a measure not influenced by oblique sectioning of the muscle and therefore less prone to measurement errors than the cross-sectional area [31]. MFD served to calculate different indicators of muscle fiber size anomalies: the variability coefficient, an indicator of abnormal variability of muscle fiber size (the normal value is below 250 for all fibers in both men and women) [31] and the atrophy and hypertrophy factors (AF and HF), indicators of the presence of an abnormal number of atrophic and hypertrophic fibers, respectively. In the *vastus lateralis* muscle, normal values for AF are below: 150 for type 1 and type 2 fibers in men, 100 for type 1 fibers in women, 200 for type 2 fibers in women. Normal values for HF are below: 150 for type 1 fibers in men, 400 for type 2 fibers in men, 400 for type 1 fibers in women and 150 for type 2 fibers in women. [31]. For each muscle section, the MFD was plotted in histograms with 10*μ*m increments to calculate the variability coefficient, AF and HF as described by Dubowitz and Sewry [31] as well as MFD average and standard deviation. Muscle sections with fewer than 100 fibers were excluded as they are not representative enough of the whole muscle [31].

#### Colocalized nuclear foci FISH and MBNL-1 immunofluorescence

Cross sections of muscle tissue were done as previously described and were left to dry at room temperature for 1 h before being stored at –80°C. When all samples were ready, they were blinded and shipped in dry ice to the University of Ottawa to complete the blinded analysis. For combined RNA FISH and MBNL1 immunofluorescence experiments, slides were air-dried for 30 min at room temperature, fixed for 30 min with 3% paraformaldehyde/PBS and permeabilized for 5 min with 0.5% Triton X-100/PBS. Slides were pre-incubated with 30% formamide, 2x saline-sodium citrate (SSC) for 10 min and incubated for 2 h at 37°C with 1 ng/*μ*l of Cy3 labeled (CAG)5 peptide nucleic acid (PNA) FISH probe (PNA Bio, #F5001) in hybridization buffer (30% formamide, 2x SSC, 0.2 mg/ml BSA, 70 mg/ml yeast tRNA, 2 mM vanadyl adenosine complex). Slides were washed for 30 min at 45°C with 30% formamide, 2X SCC followed by 2 washes of 30 min at room temperature with 1X SCC, and 3 washes of 10 min in 1X PBS. Next, slides were blocked with 1% goat serum and 1% BSA for 1 hour at room temperature. Slides were incubated overnight at 4°C with anti-MBNL1 rabbit polyclonal antibody diluted in PBS (Abcam, #ab45899, 1:250). Slides were rinsed 3 times 15 min with PBS, incubated with Alexa-488 conjugated goat anti-rabbit secondary antibody antibodies (Thermo Fisher Scientific, #A-11034, 1:200). After 3 washes of 15 min with PBS, slides were mounted with Vectashield antifade mounting medium with DAPI (Vector Laboratories, #H-1200). Fluorescent images were acquired with an Axio imager M2 microscope (Carl Zeiss) equipped with a 40X EC-Plan-Neofluar 1.3 NA oil objective lens (Carl Zeiss) and with an AxioCam mRm CCD camera (Carl Zeiss). Images were processed with the Zen Blue 3.7 software (Carl Ziess). The representative image was post-processed using deconvolution (Deblurring, Blue channel (nuclei): Strength 0.8, blur radius 12, sharpness 0.0, Green channel (MBNL1): Strength 0.7, blur radius 12, sharpness 0.0, Red channel (RNA foci): Strength 0.7, blur radius 12, sharpness 0.0). Muscle cross sections from a healthy subject were used as negative controls. Quantitative analyses were performed with the Imaris image analysis software (Oxford Instruments) as previously described [26]. In Imaris, images from one DM1 and one healthy control biopsy were used to establish foci detection parameters. The “identify spots” tool was utilized, and thresholds were adjusted for each channel until positive signal was detected in DM1 samples but not in healthy control ones. Spot size parameters were left on “auto adjust” to compensate for spot intensity. After threshold cut-off settings were set for each channel, all images were processed in batch using the same parameters. Following spot detection, each image was analyzed individually to confirm correct spot identification. Next, the “colocalization spots” tool was used in the green (MBNL1) and red (RNA Foci) channels to detect overlapping signals with a distance less than 2.5*μ*m. Note that using these experimental parameters, overlapping RNA foci/MBNL1 signal was sometimes observed in the cytoplasm, representing either cytoplasmic aggregates or nuclear aggregates with nuclei in a different focal plan. In this study, we focussed on nuclear aggregates and the percentages of nuclei positive for both RNA foci and MBNL1 were determined for analysis. Three regions of interest (ROI) per biopsy, and 3-4 images/ROI containing 11 to 66 nuclei/image were analyzed.

#### Immunoblots

Frozen skeletal muscle from biopsies with sufficient leftover mass were shipped to McGill University on dry ice. Approximately 10 mg were homogenized in an ice-cold lysis buffer A (50 mM Hepes, 150 mM NaCl, 100 mM NaF, 5 mM EDTA, 0.5% Triton X-100, 0.1 mM DTT, 2 *μ*g/ml leupeptin, 100 *μ*g/ml PMSF, 2 *μ*g/ml aprotinin, and 1 mg/100 ml pepstatin A, pH 7.2) using Mini-beadbeater (BioSpec Products) with a ceramic bead at 60 Hz. Muscle homogenates were kept on ice for 60 min with periodic agitation and then were centrifuged at 5000 g for 15 min at 4°C, supernatants were collected, and pellets were discarded. The protein content in each sample was determined using the Bradford or BCA (Pierce) method. Aliquots of crude muscle homogenates were mixed with Laemmli buffer (6×, reducing buffer, # BP111R, Boston BioProducts) and subsequently denatured for 5 min at 95°C. Equal amounts of protein extracts (20 *μ*g per lane) were separated by SDS-PAGE, and then transferred onto polyvinylidene difluoride (PVDF) (Bio-Rad Laboratories) using a wet transfer technique. The total proteins on membranes were detected with stain-free technology from Bio-Rad. Membranes were blocked in PBS + 1% Tween^®^ 20 + 5% bovine serum albumin (BSA) for 1 hour at room temperature and then incubated with the specific primary antibodies for 1 hour at room temperature or overnight at 4°C. The complete list of antibodies used for immunoblots analysis can be found in supplementary information Table 1 (glycogen synthase kinase-3 beta [GSK3*β*], LC3 [LC3BI and LC/B11], p62 and oxidative phosphorylation [OXPHOS] proteins). All antibodies were diluted in blocking buffer. Immunoreactivity was detected using enhanced chemiluminescence substrate (Biorad, Clarity ECL substrate, 170–5060) with the ChemiDoc™ Imaging System. The optical densities (OD) of protein bands were quantified using ImageLab 6.1 software (Bio-Rad Laboratories) and normalized to the intensity of the stain-free (SF) blot image of the corresponding sample.

#### Statistical analysis

The formula used for fold change was P4/P3. All muscle fiber size indicators fold change (MFD for all fibers, type 1 fibers and type 2 fibers, type 1 and type 2 AF, type 1 and type 2 HF and variability coefficient for all fibers) were correlated with maximal muscle strength fold change of the biopsied leg using Spearman’s correlation as this correlation is nonparametric. Fold change of percentage of nuclear foci colocalized with MBNL1 positive nuclei (foci/MBNL1+) was correlated with age, sex, phenotype, CTG repeats at P3, fold change of maximal strength of the biopsied knee extensors, type of physical activity practiced over the 3 years between the evaluations and percentage of nuclear foci colocalized with MBNL1 at P3 (baseline) using Spearman’s correlations as a prior step to linear regression analysis. Correlations with a *p*-value <0.2 were included in the stepwise regression model analysis as this specific analysis was exploratory. To allow for a stepwise linear regression model, categorical variables with more than two categories had some categories grouped so only two categories remained (for phenotypes: infantile and juvenile were grouped together while adult and late were grouped together, for the type of physical activity: strength training vs. others). To identify possible biomarkers in protein expression, as with histomorphology indicators, the fold change of the immunoblot analysis were correlated with the fold change of maximal strength of the biopsied leg using Spearman’s correlations. All correlations were evaluated according to the following criteria: <0.25 – little or no relationship; 0.25–0.50 fair relationship; 0.50–0.75 moderate to good relationship and >0.75 good to excellent relationship [32]. All statistical analyses were completed using IBM SPSS Statistics 19 (IBM, Armonk, USA).

## RESULTS

### Participants

A total of 22 participants underwent/completed both muscle biopsies and clinical evaluations at P3 and P4. Some cross-sectional cuts were not suited for either the fiber size and typology analysis or nuclear foci and MBNL1 colocalization analysis. Furthermore, only the participants with sufficient biological material were included in the immunoblotting experiments. Participants’ characteristics are presented in Table 1. A total of 17 participants were included in the fiber typing and size protocol, 21 in the nuclear foci/MBNL1 co-localization protocol and 11 in the immunoblotting protocol. Participants’ characteristics for individual protocols can be found in supplementary information Tables 2 to 4.

**Table 1:** Participants’ characteristics

	Sex		Phenotype				Physical activity type		
	Women	Men	Infantile	Juvenile	Adult	Late	Sedentary	Physical activity	Strength training
Number (%)	11 (50)	11 (50)	1 (5)	7 (32)	10 (45)	4 (18)	7 (32)	9 (41)	6 (27)
Age (SD) [min-max]	49 (6) [27-65]	50 (12) [29-64]	33 (–) [33-33]	43 (12) [27-57]	52 (10) [29-62]	60 (5) [54-65]	55 (4) [47-60]	46 (14) [27-65]	48 (13) [29-64]
CTG (SD) [min-max]	648 (318) [101-1097]	431 (283) [85-954]	675 (–) [675-675]	742 (206) [570-1097]	563 (280) [170-1097]	91 (7) [85-101]	673 (392) [90-1097]	543 (266) [101-954]	378 (244) [85-608]
Number ♀/♂	–	–	1/0	5/2	4/6	1/3	4/3	7/2	0/6
Number infantile/ juvenile/ adult/ late	–	–	–	–	–	–	0/2/4/1	1/4/3/1	0/1/3/2

Abbreviations: %: percentage, SD: standard deviation, min: minimum, max: maximum, ♀: women, ♂: men.

### Fiber typing and size

**Fig. 1:**
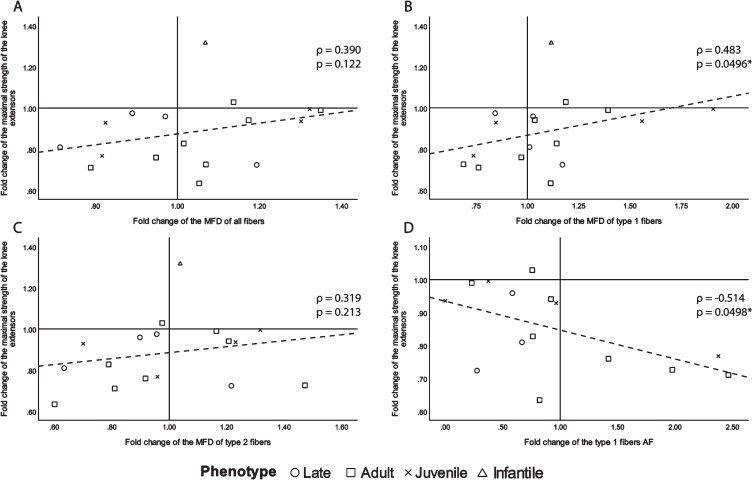
Scatter plots of the fold change of the maximal strength of the knee extensors of the biopsied leg with fiber size and abnormal size indicators with *ρ*> 0.250. A- fold change of all fibers MFD, B- fold change of type 1 fiber MFD, C- fold change of type 2 fibers MFD and D- fold change of type 1 fibers AF. *ρ*: Spearman’s rank correlation coefficient, p: *p*-value, MFD: minimal Feret’s diameter, AF: atrophy factor, ^*^p-value <0.050.

Since muscle atrophy is a highly detrimental manifestation of DM1 [13], we first investigated changes in muscle fiber size to document its evolution with the progression of the disease. To do so, MFD and AF were chosen as the most relevant variables, and only those that correlate with maximal strength changes with *ρ*> 0.250 are presented in Fig. 1. Other correlations (correlations *ρ* < 0.250 for AF and correlations for HF and variability coefficient) can be found in supplementary information, Table 5. There was a moderate to good inverse correlation between the type 1 fibers AF change and maximal muscle strength change, which was also statistically significant (*ρ*= –0.514, *p* = 0.0498, Fig. 1D). There was also a fair significant correlation between the fold change of maximal muscle strength and the fold change of MFD of type 1 fibers (*ρ*= 0.483, *p* = 0.0496, Fig. 1B). For the sake of transparency, although not significant, the following fair or above correlations are shown. All fibers and type 2 fibers MFD change showed a fair correlation with maximal muscle strength change (*ρ*= 0.390, *p* = 0.122 and *ρ*= 0.319, *p* = 0.213 respectively, Fig. 1A and C). A positive correlation between muscle strength and fiber MFD indicates that the greater maximal strength decrease over time was accompanied by greater decreases in fiber size. The strongest correlation being with type 1 fibers, which is very relevant in DM1, as the disease has a preferential atrophy for type 1 fibers [31]. The negative correlation between muscle strength and type 1 AF further demonstrates that the higher the fold change of the atrophy factor (the more the quantity of type 1 atrophic fibers increased) the more the maximal strength of the biopsied knee extensor decreased.

### Colocalized nuclear foci and MBNL1

**Fig. 2:**
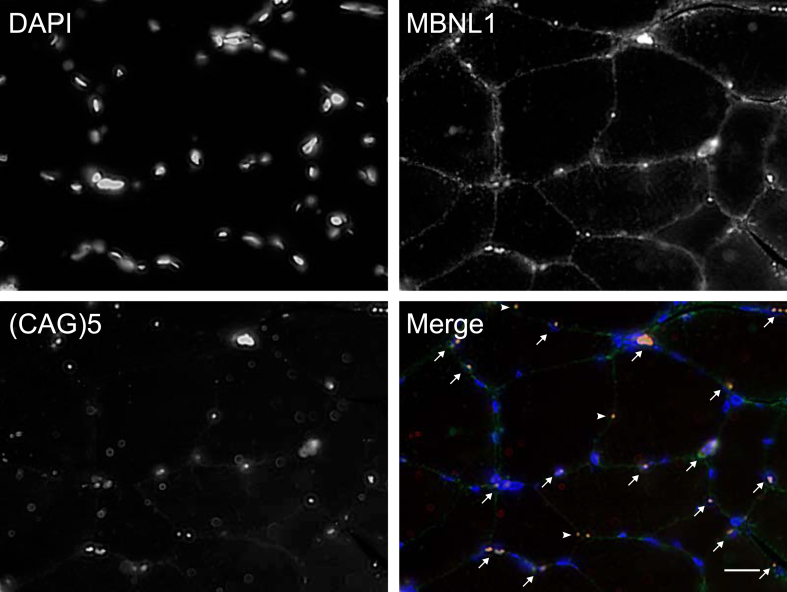
Representative image of colocalized nuclear foci FISH and MBNL-1 immunofluorescence. Scale bar = 20*μ*m. DAPI showing myonuclei, MNBL1, (CAG)5 RNA FISH showing nuclear foci and Merge showing a composite image (DAPI in blue, MBNL1 in green and RNA foci in red). Arrows point to RNA foci/MBNL1 positive nuclei. Note that some RNA foci/MBNL1 aggregates did not colocalize with DAPI (arrowheads).

**Fig. 3:**
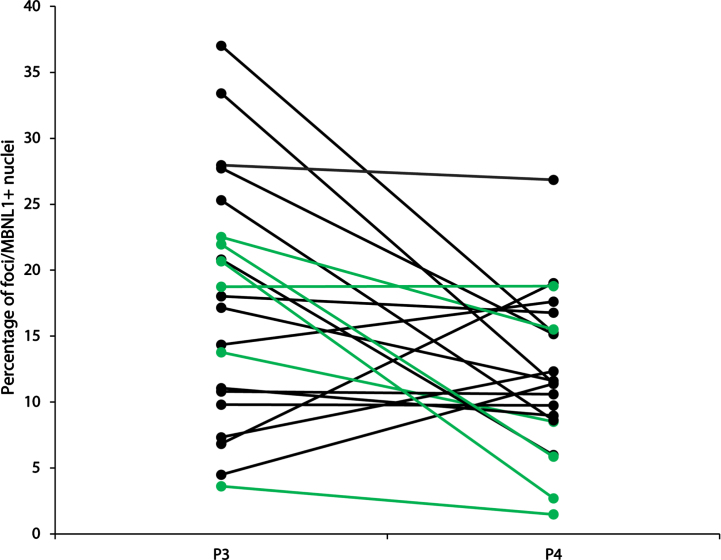
Percentage of foci/MBNL+ nuclei at P3 and P4. In green: participants who participated in a strength training program between P3 and P4. In black: all other participants.

The accumulation of nuclear foci and sequestration of MBNL-1 is acknowledged as the major physiopathological mechanism in DM1 [13]. Accordingly, we next investigated how the accumulation of nuclear foci with MBNL1 changes over time. To this end, we performed a combined RNA FISH and MBNL1 immunofluorescence staining on muscle cross-sections, and quantified, in a blind manner, the percentage of nuclei positive for both RNA foci and MBNL1 aggregates (Fig. 2 and supplementary information Fig. 1). This dual detection method ensures that only positive nuclei exhibiting both overlapping RNA foci and MBNL1 aggregates are identified, thereby minimizing the potential for overestimation resulting from non-specific signals compared to single detection methods. Surprisingly, many participants presented a decreased percentage in doubly labeled foci/MBNL1+ nuclei. Figure 3 shows a visual representation of the foci/MBNL+ nuclei percentage change between P3 and P4 for each individual. Table 2 shows a descriptive presentation of the subjects with either increased, unchanged or increased the percentage of nuclear foci colocalized with MBNL-1 with a 20% change cut-off. This cut-off value was chosen as the standard deviation within the participants ranged from 2.0% to 25.8%, with an average of 8.5% (data not shown). Therefore, to ensure that the observed change was a real change, the conservative value of 20% was chosen. Interestingly, among the 6 participants who underwent strength training between the two time points, 5 of them had a decreased percentage of positive nuclei. The 6th participant did not show changes in foci/MBNL1+ nuclei accumulation.

**Table 2:** Descriptive data of subjects according to their change in foci/MBNL1+ nuclei percentage

Variable /group	Increased	Unchanged	Decreased
Number	4	6	11
Baseline age±SD	43±15	48±12	52±11
CTG±SD	735±246	623±209	391±334
Sex	4♀	3♀ / 3♂	3♀ / 8♂
Baseline (P3) % of nuclei positive for nuclear foci and MBNL1±SD	8.26±4.25	16.07±6.99	22.18±9.11
MIRS score 1/2/3/4/5	0/1/1/2/0	0/0/2/4/0	0/2/2/7/0
Phenotype
Infantile, *n* (detailed n)	1 [100%] (1 ♀)	0 [0%]	0 [0%]
Juvenile, *n* [%] (detailed n)	2 [29%] (2 ♀)	4 [57%] (3 ♀, 1 ♂)	1 [14%] (1 ♂)
Adult, *n* [%] (detailed n)	1 [11%] (1 ♀)	2 [22%] (2 ♂)	6 [67%] (2♀, 4 ♂)
Late, *n* [%] (detailed n)	0 [0%]	0 [0%]	4 [100%] (1♀, 3♂)
Physical activity type
Sedentary, *n* [%] (detailed n)	1 [17%] (1♀J)	1 [17%] (1♀ J)	4 [68%] (1♀A, 2♂A, 1♂L)
Physical activity, *n* [%] (detailed n)	3 [33%] (1♀I, 1♀J, 1♀A)	4 [45%] (2♀J, 1♂J, 1♂A)	2 [22%] (1♀A, 1♀L)
Strength training, *n* [%] (detailed n)	0 [0%]	1 [17%] (1♂A)	5 [83%] (1♂J, 2♂A, 2♂L)

Abbreviations: SD: standard deviation, n: number, %: percentage, detailed n: number of each sex and phenotype within the total number of that case, ♀: women, ♂: men, I: Infantile, J: Juvenile, A: Adult, L: Late. The cut-off fold change to distinguish the groups is 20%, meaning that the increased group had an increase >20% fold change, the unchanged group had <20% fold change increase or decrease and the decreased group had >20% decrease.

As a next step, and prior to linear regression, Spearman’s correlation was used to find participants’ characteristics that were linked to the change in the percentage of foci/MBNL1+ nuclei. For regression analysis, some categorical data (phenotype and physical activity type) were converted to contain only two categories as the linear regression model only allowed for binomial categorical data (Table 3). Therefore, we grouped the phenotypes that have the most clinical similarities: infantile and juvenile DM1 participants were grouped together, while adult and late onset created the other group. For the physical activity type, strength training was chosen to be considered its own group because we hypothesized that this type of training is of sufficient intensity to influence nuclear foci accumulation and MBNL1 sequestration [33]. Fold change in the percentage of foci/MBNL1+ nuclei correlations with different variables can be found in Table 3.

**Table 3:** Spearman’s correlations with the fold change of foci/MBNL1+ nuclei

	Spearman’s *ρ*	*p*-value
Continuous data
Age	–0.199	0.387
CTG at baseline (P3)	0.299	0.187^*^
Fold change of maximal strength of the biopsied knee extensor	–0.028	0.909
Percentage of foci/MBNL1+ nuclei at baseline (P3)	–0.565	0.008^*^
Categorical data
Sex	0.472	0.031^*^
Phenotype
Infantile and juvenile vs. adult and late	0.551	0.010^*^
Physical activity type
Strength training vs. others	0.348	0.122^*^

^*^significant correlations for the stepwise correlation model where *p*-value <0.2.

The significant correlations (*p* < 0.2) were then run in the stepwise linear regression model. The best model had an adjusted R square of 0.441 (*p* = 0.002) with two significant variables (Table 4): 1) Percentage of nuclei containing nuclear foci and MBNL1 aggregates at P3 and 2) physical activity type in two categories, strength training vs. others. This shows that together, these two variables explain 44.1% of the fold change in the percentage of foci/MBNL1+ nuclei over time. The standardized beta represents the strength of the effect of the variable in the model, meaning that the percentage of foci/MBNL1+ nuclei at P3 has 1.6 times more effect than the participation in strength training in the model. Being a continuous variable, the percentage of foci/MBNL1+ nuclei at P3’s beta represents the average multiplier, meaning that on average, the percentage of foci/MBNL1+ nuclei at P3 is multiplied by –0.046 to obtain the fold change in foci/MBNL1+ nuclei. While participating in strength training is a categorical variable, its beta represents the average difference, in other words, the strength training group had an average difference in foci/MBNL1+ nuclei fold change of –0.572 compared to the other participants. In summary, the higher value of the percentage of foci/MBNL1+ nuclei at P3 and the participation in a strength training program led to a reduction in the percentage of foci/MBNL1+ nuclei over time. These data are encouraging, as they suggest a plateau effect in foci/MBNL1+ nucleus accumulation. It is also possible that strength training contributes to the degradation of foci/MBNL1+ nuclei.

**Table 4:** Linear regression model for foci/MBNL1+ nuclei change over 3 years

Adjusted R^2^	Model F value	Model *p*-value	Independent variables	Beta	95% CI	Variable F value	Variable *p*-value	Standardized Beta
0.441	8.884	0.002	Intercept	1.875	1.318; 2.431	39.232	<0.001
	Percentage of foci/MBNL1+ nuclei at P3	–0.046	–0.073; –0.020	13.599	0.002	–0.618
	Participation in a strength training program	–0.572	–1.102; –0.042	5.149	0.036	0.380

Abbreveation: CI : Confidence interval

### Immunoblots

As described in the introduction, several proteins are misregulated in DM1 muscle and contribute to the underlying physiopathology. Among them, proteins we hypothesized had an impact on muscle wasting, such as GSK3*β* and autophagy markers (p62, LC3) [13]. In addition, recent studies have also highlighted significant defects in mitochondria from DM1 muscle [16]. Here, we therefore examined the expression levels of several key proteins relevant to the DM1 physiopathology to not only complement our morphological and localization analyses (see above) but to also determine their potential as valid biomarkers of disease progression. Representative immunoblot images can be found in supplementary information Fig. 2.

**Fig. 4:**
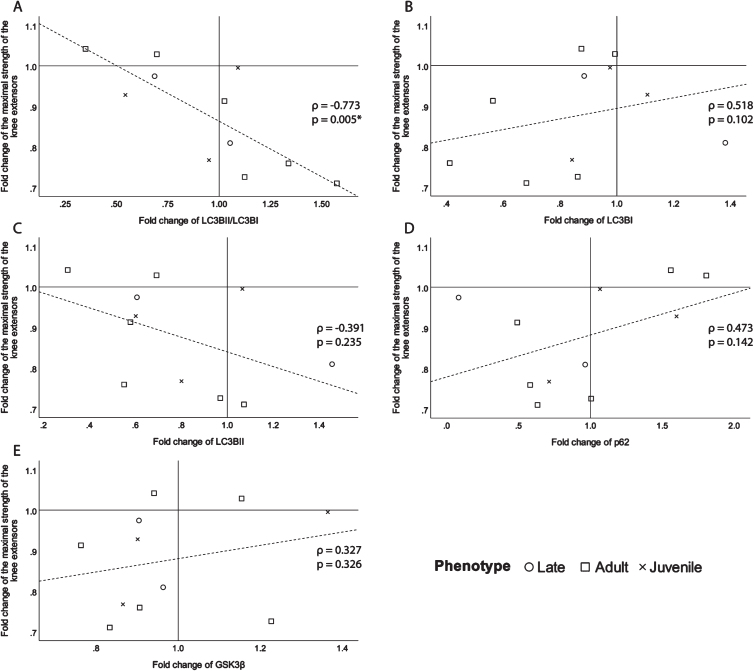
Scatter plots of the fold change of the maximal strength of the knee extensors of the biopsied leg with A-the fold change of the ratio of LC3BII/LC3BI expression, B- the fold change of LC3BI expression, C- the fold change of LC3BII expression, D- the fold change of p62 expression and E-the fold change of GSK3*β* expression. *ρ*: Spearman’s rank correlation coefficient, p: *p*-value, ^*^p-value <0.050.

To identify their potential as biomarkers, the fold changes of the selected proteins were correlated with the fold change of the maximal strength of the knee extensors of the biopsied leg (Fig. 4). There is a significant good to excellent correlation coefficient (*ρ*= –0.773, *p* = 0.005, Fig. 4A) with the ratio of LC3BII/LC3BI. This signifies the more the ratio of LC3BII/LC3BI increases over time, meaning that autophagy increases over time, the more the maximal strength of the knee extensors decreases. The following correlations, although not significant still had a fair or better correlation strength and were presented for transparency and clarity. There is a moderate to good relationship (*ρ*= 0.518, *p* = 0.102, Fig. 4B) between the fold change of LC3BI and maximal strength fold change. There is also a fair inverse correlation (*ρ*= –0.391, *p* = 0.235, Fig. 4C) between the fold change of LC3BII, which is an indicator of the quantity of autophagosomes, and the change in maximal strength. The autophagy indicator, p62, shows a fair relationship (*ρ*= 0.473, *p* = 0.142, Fig. 4D) with maximal strength change. Since p62 levels reduce when autophagy is induced [34], it makes sense that the correlation with maximal strength change is positive. A fair relationship between maximal strength fold change and GSK3*β* (*ρ*= 0.327, *p* = 0.326, Fig. 4E), has been found. The positive direction of the correlation means that the more GSK3*β* decreases over time, the more maximal strength decreases. The correlations of maximal muscle strength fold change with OXPHOS proteins expression fold change were all *ρ*<0.25 (supplementary information Table 6).

## DISCUSSION

The aim of this study was to identify physiopathological markers related to the maximal strength loss over time in individuals with DM1. To achieve this, muscle biopsies of the vastus lateralis were collected 3 years apart within a larger longitudinal study. To our knowledge, this is the first longitudinal study in DM1 to analyze skeletal muscle biopsies for physiopathological markers such as fiber size and foci/MBNL1+ nuclei. Thus, this represents an important first step for the identification of biomarkers that are essential to inform clinical trials and monitor disease progression. The chosen variables of interest in this study were muscle fiber histomorphology, nuclear foci with MBNL-1 colocalization and protein expression of some key proteins that are misregulated in DM1.

Our results have shown that the fold change of maximal strength of the biopsied muscle show fair to good correlations with the fold change of all, type 1 and type 2 MFD as well as with the AF of type 1 fibers. This indicates that the progression of fiber atrophy, especially type 1 fibers, is an associated factor to maximal muscle loss. These results are not surprising as DM1 is known to present a preferential type 1 muscle fiber atrophy [31]. Additionally, these correlations are significant even if there is no significant change of the average of MFD and AF over three years while the maximal muscle strength has significantly decreased (data not shown). This can be explained by examining the scatter plots in Fig. 1. While we can see that almost all participants have decreased maximal strength of the knee extensors of the biopsied leg (fold change <1), some participants showed an increase in type 1 MDF and a decrease in type 1 AF (Fig. 1). Nevertheless, the participants who showed an increase in type 1 MDF and a decrease in type 1 AF are the ones who lost the least strength. This suggests that there are other factors not explained by muscle fiber size, that contribute to maximal strength loss in DM1. It also suggests that an increase in type 1 MFD/reduction in type 1 AF has a protective effect on maximal strength loss. It does seem that muscle fiber hypertrophy might be a protective mechanism in DM1, as it has been reported that in the increasingly advanced stages of DM1, there is a presence of great heterogeneity of muscle fiber size, with many atrophic fibers, alongside hypertrophic ones [31]. Clearly, additional studies are warranted to gain further insight into the driving factors contributing to maximal muscle strength loss in DM1. Furthermore, this highlights the importance of individual assessments in DM1, given the high heterogeneity of the disease, as some changes might be missed if only averages of whole participants’ groups are measured.

The next step of this study was to evaluate the accumulation of nuclear foci with MBNL1. Surprisingly, many participants had a decrease in their percentage of foci/MBNL1+ nuclei. To confirm which variables had an influence on foci/MBNL1+ nuclei reduction, a regression model was used. Of course, this model is exploratory in nature, and the low number of participants does not allow for a complete explanatory model. As expected, correlation results showed phenotype and strength training were significant variables, as well as CTG repetitions, age, and baseline percentage of foci/MBNL1+ nuclei. Surprisingly, even if it has already been shown that with a reduction CUG expansion (CUG^exp^) transcripts or MBNL1 sequestration, there is a reduction of DM1 symptoms [35], foci/MBNL1+ nuclei change did not correlate with maximal muscle strength change. The stepwise regression model found that using baseline percentages of foci/MBNL1+ nuclei with participation in strength training was the best model. Indeed, this model would explain 44.1% of the variance of foci/MBNL1+ nuclei percentage, where the percentage of foci/MBNL1+ nuclei at baseline has more impact than participation in a strength-training program. The effect of exercise in foci/MBNL1+ nuclei reduction is not surprising, as it has already been reported in mouse models [26]. However, this confirmation in humans is key as it clearly illustrates the potential benefits of regular exercise training on the DM1 muscle pathology. Nevertheless, strength training as a key variable in this regression model reinforces the growing body of literature that strength training has an important role in the management of muscular impairments in DM1 [8, 21–24, 36–38]. As previously stated, the importance of the percentage of foci/MBNL1+ nuclei at baseline as a variable in this regression model suggests that there may be a plateau effect in foci/MBNL1+ nucleus accumulation. Another unexpected result was that the participants with decreased percentage of foci/MBNL1+ nuclei had the highest baseline percentage of foci/MBNL+ nuclei and were mostly of late and adult phenotype and with the lowest CTG repetitions. This counterintuitive result as well as the plateau effect brought us to the hypothesis that nuclear foci dynamics might come into play in unexpected ways. Previous studies have shown that RNA foci are dynamic structures [39]. There have indeed been multiple mechanisms that influence foci stability and cytoplasmic export as well as MBNL1 sequestration [12] that could contribute to this plateau effect. Notably, the role of RNA-binding proteins, including DEAD-box helicases, has been found to play an important role in nuclear foci stability [12]. It has also been reported that cell cycles influence foci/MBNL1+ nuclei accumulation, and that mitosis can decrease foci/MBNL1+ nuclei load [40]. This could be a mechanism induced by strength training that helps reduce foci/MBNL1+ nuclei load when satellite cells replicate to increase myonuclei numbers and sustain muscle growth.

Finally, to evaluate the underlying cellular mechanisms and identify a potential marker that could accurately follow disease progression, the fold change in selected protein expression was correlated with the change in maximal strength. The most interesting result is the strong and significant negative correlation between the fold change in LC3BII/LC3BI ratio and maximal strength. Along with the fair negative correlation of the fold change of LC3BII with the change in maximal strength of the biopsied knee extensor, the latter result brings some insight for biomarker identification. An increase in LC3BII/LC3BI ratio and LC3BII are indicators of increased autophagy, and impaired autophagic flux has been linked to muscle wasting in DM1 [41]. This link between the progression of maximal strength loss and autophagy is further reinforced by the fair positive correlation between the fold change of p62 and the fold change of maximal muscle strength. Indeed, the induction of autophagy decreases p62 levels by degrading p62, if there is no transcriptional change [34]. Therefore, the positive direction of this correlation, is not surprising and means that the more its expression decreases over time, the more autophagy increases, the more maximal strength decreases. It is to be noted that the correlations of change in maximal muscle strength with changes in LC3BII and p62 are only fair and should be interpreted with caution. They serve to support the results from the good to excellent correlation of maximal muscle strength change with LC3BII/LC3BI and point to the role of autophagy in skeletal muscle impairment progression in DM1. Furthermore, these results support emerging literature that there are indeed autophagy impairments that are to be considered in DM1 physiopathology [15, 18]. It is not surprising that there were no correlation coefficients above 0.25 for the change in OXPHOS protein expression with maximal muscle strength, as although reduced mitochondrial content is increasingly reported in DM1, it has not been associated with muscle weakness [16, 17]. These results remain exploratory and serve to guide future research.

### Study limitations

The main limitation of this study is the small sample size, which increases the risk of type II errors and limits generalizations to the whole DM1 population. This is especially true for the immunoblot analysis. The small sample size also limits regression models that would help determine important variables that explain the observed changes in skeletal muscle. Furthermore, our cohort of participants is somewhat heterogenous and includes various phenotypes (all adult-onset phenotypes except for one participant) and both sexes, two known factors that modify DM1 clinical presentation [5, 6]. The diversity within the studied population might have led to overlooked findings that could have held significance for a particular phenotype or sex. This heterogeneity was deliberate and was made to address the limitations posed by the small sample size. Nevertheless, this study represents the most extensive exploration of skeletal muscle physiopathology in DM1 conducted longitudinally to date.

## CONCLUSION

This is the first study, to our knowledge, that analyzes skeletal muscle biopsies with a longitudinal design in humans with DM1. It places important first steps to understanding how skeletal muscle evolves with the progression of the disease. The main findings of this study are the link between type 1 fiber size change with maximal muscle strength change as well as the importance of the baseline percentage of foci/MBNL+ nuclei and participation in a strength training program in foci/MBNL1+ nuclei percentage change over time. These last results suggest a plateau effect for foci/MBNL+ nuclei percentage and add to the building evidence of the importance of exercise and strength training in the management of DM1. Results indicate that autophagy, notably LC3BII/LC3BI ratio, could be an interesting cellular mechanism to study skeletal muscle impairment in DM1, further studies are needed to confirm this.

## Supplementary Material

Supplementary Material

## Data Availability

Data can be available upon reasonable requests to the corresponding author.
