# Supplementary material for: Changes in Physiopathological Markers in Myotonic Dystrophy Type 1 Skeletal Muscle: A 3-Year Follow-up Study
Source: J Neuromuscul Dis. 2024 Sep 3;11(5):981–95. doi: 10.3233/JND-230139 (PMC11380274; doi:10.3233/JND-230139)
Supplement: Supplementary Material [file jnd-11-jnd230139-s001.docx]

Supplemental information

Table 1: Antibodies used for immunoblots.

| **Antibody** | **Source / Product no.** | **Dilution** |
| --- | --- | --- |
| GSK3-β | Santa cruz, # 71186 | 1/1000 |
| anti-OXPHOS | Abcam / MitoSiences # ab110413 | 1/1000 |
| Rabbit anti-LC3 | Cell signaling #12741 | 1/1000 |
| Mouse anti-p62/SQSTM1 | Novus Biologicals Inc. clone 2C11 | 1/1000 |
| Goat anti mouse IgG | Abcam # Ab6728 | 1/5000 |
| Goat anti rabbit IgG | Abcam # Ab6721 | 1/5000 |

Table 2: Participant’s characteristics: histomorphological data.

|  | **Sex** | | **Phenotype** | | | | **Physical activity type** | | |
| --- | --- | --- | --- | --- | --- | --- | --- | --- | --- |
|  | **Women** | **Men** | **Infantile** | **Juvenile** | **Adult** | **Late** | **Sedentary** | **Physical activity** | **Strength training** |
| Number (%) | 8 (47) | 9 (53) | 1 (6) | 4 (24) | 8 (47) | 4 (24) | 6 (35) | 7 (41) | 4 (24) |
| Age (SD) [min-max] | 47 (13) [27-65] | 54 (8)  [39-64] | 33 (--) [33-33] | 40 (12) [27-57] | 54 (5)  [45-62] | 60 (5)  [54-65] | 56 (5)  [48-64] | 48 (15) [27-65] | 50 (9)  [39-58] |
| CTG (SD) [min-max] | 549 (309) [101-1097] | 342 (238) [85-673] | 675 (--) [675-675] | 598 (20) [570-615] | 505 (298) [170-1097] | 91 (7) [85-101] | 470 (398) [89-1097] | 455 (225) [101-675] | 369 (244) [85-608] |
| Number ♀/♂ | -- | -- | 1/0 | 3/1 | 3/5 | 1/3 | 2/4 | 6/1 | 0/4 |
| Number infantile/ juvenile/ adult/ late | -- | -- | -- | -- | -- | -- | 0/1/3/2 | 1/2/3/1 | 0/1/2/1 |

Table 2 legend: %: percentage, SD: standard deviation, ♀: women, ♂: men

Table 3: Participant’s characteristics: nuclear foci and MBNL-1 co-localization

|  | **Sex** | | **Phenotype** | | | | **Physical activity type** | | |
| --- | --- | --- | --- | --- | --- | --- | --- | --- | --- |
|  | **Women** | **Men** | **Infantile** | **Juvenile** | **Adult** | **Late** | **Sedentary** | **Physical activity** | **Strength training** |
| Number (%) | 10 (48) | 11 (52) | 1 (5) | 7 (33) | 9 (43) | 4 (19) | 6 (28) | 9 (43) | 6 (28) |
| Age (SD) [min-max] | 48 (12) [27-65] | 50 (12) [29-64] | 33 (--) [33-33] | 43 (12) [27-57] | 51 (10) [29-62] | 60 (5)  [54-65] | 54 (3) [47-57] | 46 (14) [27-65] | 48 (13) [29-64] |
| CTG (SD) [min-max] | 624 (325) [101-1097] | 431 (283) [85-954] | 675 (--) [675-675] | 742 (206) [570-1097] | 527 (271) [170-1097] | 91 (7)  [85-101] | 638 (417) [90-1097] | 543 (266) [101-954] | 378 (244) [85-608] |
| Number ♀/♂ | -- | -- | 1/0 | 5/2 | 3/6 | 1/3 | 3/3 | 7/2 | 0/6 |
| Number infantile/ juvenile/ adult/ late | -- | -- | -- | -- | -- | -- | 0/2/3/1 | 1/4/3/1 | 0/1/3/2 |

Table 3 legend: %: percentage, SD: standard deviation, ♀: women, ♂: men.

The control subject used as negative control for this protocol was a healthy 31-year-old female who practiced moderate physical activity.

Table 4: Participant’s characteristics: Immunoblotting

|  | **Sex** | | **Phenotype** | | | | **Physical activity type** | | |
| --- | --- | --- | --- | --- | --- | --- | --- | --- | --- |
|  | **Women** | **Men** | **Infantile** | **Juvenile** | **Adult** | **Late** | **Sedentary** | **Physical activity** | **Strength training** |
| Number (%) | 5 (45) | 6 (55) | 0 (0) | 3 (28) | 6 (54) | 2 (18) | 3 (28) | 4 (36) | 4 (36) |
| Age (SD) [min-max] | 47 (14)  [27-60] | 57 (7)  [45-64] | -- | 40 (15)  [27-57] | 56 (6)  [45-62] | 60 (5)  [57-64] | 57 (2)  [55-60] | 46 (16)  [27-62] | 56 (8)  [45-64] |
| CTG (SD) [min-max] | 648 (134)  [567-885] | 302 (195)  [85-533] | -- | 595 (23)  [570-615] | 515 (217)  [254-885] | 87 (3)  [85-89] | 585 (316)  [254-885] | 560 (42)  [500-601] | 264 (218)  [85-533] |
| Number ♀/♂ | -- | -- | -- | 3/0 | 2/4 | 0/2 | 2/1 | 3/1 | 0/4 |
| Number infantile/ juvenile/ adult/ late | -- | -- | -- | -- | -- | -- | 0/1/2/0 | 0/2/2/0 | 0/0/2/2 |

Table 4 legend: %: percentage, SD: standard deviation, ♀: women, ♂: men.

Table 5: Other correlations between fold change of maximal muscle strength of the knee extensors with fold change of muscle fiber size and abnormal size indicators.

| **Variable** | **Spearman’s correlation coefficient with the fold change of the maximal strength of the knee extensors** | **p-value** |
| --- | --- | --- |
| Fold change of the variability coefficient | -0.120 | 0.6461 |
| Fold change of type 1 fibers HF | 0.288 | 0.3176 |
| Fold change of type 2 fibers AF | -0.239 | 0.3904 |
| Fold change of type 2 fibers HF | 0.343 | 0.2296 |

Fig. 1: Representative images of colocalized nuclear foci FISH and MBNL-1 immunofluorescence.


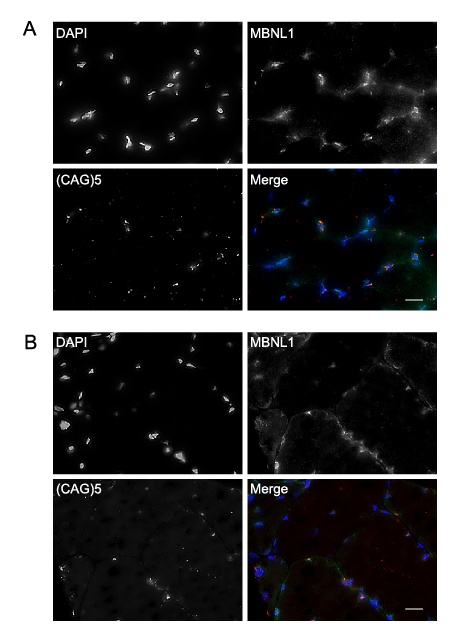


Fig. 1 legend: Two other representative image sets of colocalized nuclear foci FISH and MBNL-1 immunofluorescence. Fig. 1 A and Fig. 1 B are from different participants. Scale bar = 20 μm. DAPI showing myonuclei in the first image, MNBL1 in the second image, (CAG)5 RNA FISH showing nuclear foci in the third image and Merge showing a composite image in the fourth image.

Fig. 2: Representative immunoblot images for all evaluated proteins.


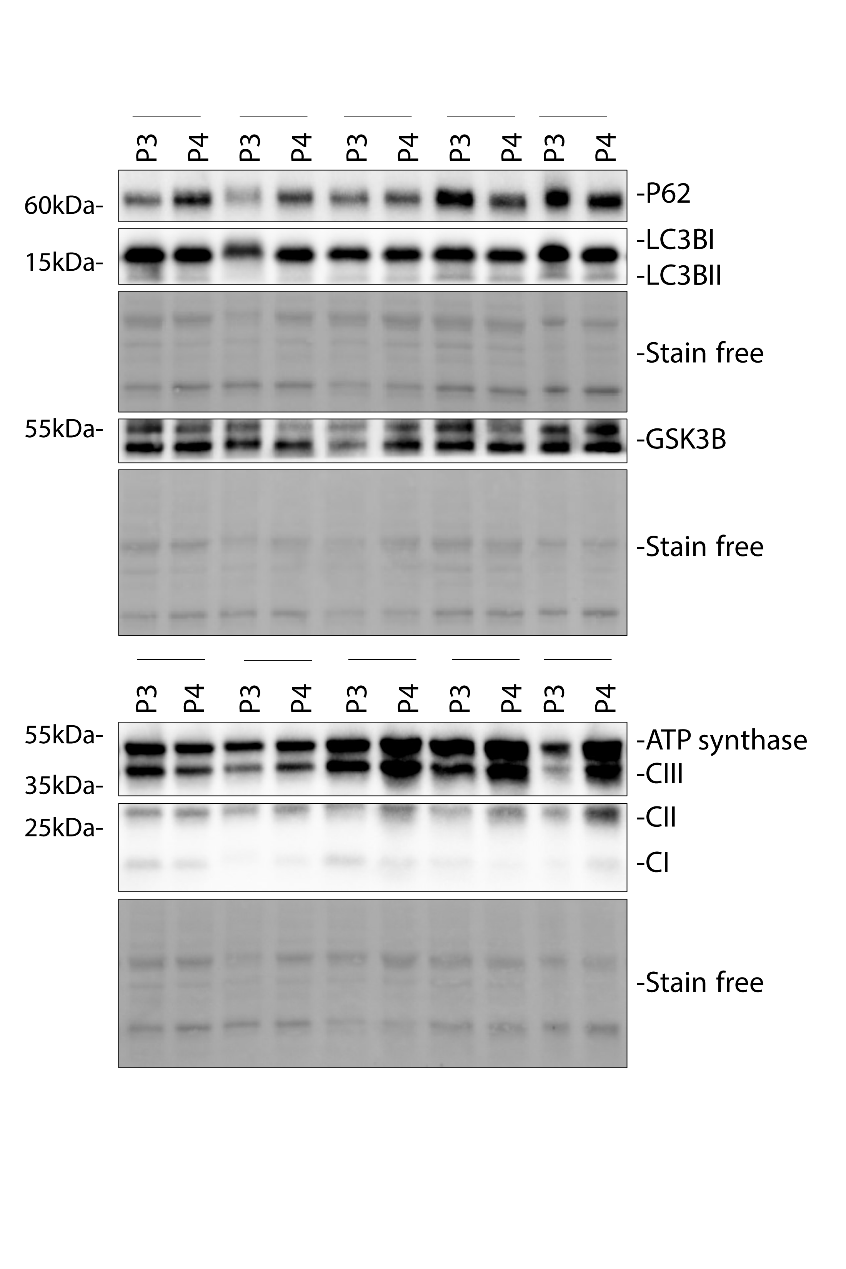


Fig. 2 legend: Representative images of Western blots participants with DM1. Western blot images and their stain-free images as the loading control (presented below their respective proteins).

Table 6: Correlations of the fold change of maximal strength of the knee extensors with the fold change of OXPHOS proteins expression

| **OXPHOS protein** | **Spearman’s correlation coefficient with the fold change of the maximal strength of the knee extensors** | **p-value** |
| --- | --- | --- |
| Complex I | 0.155 | 0.650 |
| Complex II | 0.018 | 0.958 |
| Complex III | 0.009 | 0.979 |
| ATP synthase | 0.045 | 0.894 |
